# Lessons Learnt From 25 Years of Endophthalmitis: Causes, Microbiology and Visual Outcome Trends

**DOI:** 10.1111/ceo.70133

**Published:** 2026-06-15

**Authors:** Zelia K. Chiu, Rosie C. H. Dawkins, Gursimrat K. Bhullar, Ebrar Al‐Yasery, Penelope J. Allen

**Affiliations:** ^1^ Vitreoretinal Unit Royal Victorian Eye and Ear Hospital East Melbourne Victoria Australia; ^2^ Centre for Eye Research Australia East Melbourne Victoria Australia; ^3^ Department of Surgery The University of Melbourne Parkville Victoria Australia

## Abstract

**Background:**

We aimed to study the trends in microbiology, precipitating causes and outcomes of endophthalmitis in Victoria, Australia, over a 25‐year period. To the best of our knowledge, this is the longest study of endophthalmitis in Australia.

**Methods:**

We conducted a prospective study of all endophthalmitis cases to the Royal Victorian Eye and Ear Hospital, a state‐wide tertiary referral centre for ocular conditions, thereby capturing nearly all state‐wide endophthalmitis cases. All patients presenting with endophthalmitis were included. Demographic data, examination findings, microbiology, management and outcomes were collected.

**Results:**

A total of 1423 eyes from 1408 patients were included. The mean (SD) age was 71.35 (16.12). The median presenting best‐corrected visual acuity was hand movements. Cataract surgery formed the plurality of annual cases until 2009; intravitreal injections exceeded following. The rate of endophthalmitis secondary to cataract surgery decreased from 0.29%–0.02% over the 25‐year period, with a significant decrease following the introduction of prophylactic cefazolin; however, the rate of endophthalmitis secondary to intravitreal injections remained relatively stable (0.01%–0.04%). 63.03% were culture‐positive; the plurality grew gram‐positive bacteria (42.94%). Visual outcomes were biphasic; 44.99% had a final BCVA of less than 6/60 (including enucleation/evisceration), while 30.6% reached 6/12 or better. Poor visual outcomes were associated with corneal infection; cataract surgery and intravitreal injections were associated with better outcomes.

**Conclusions:**

This study demonstrated declining rates of endophthalmitis secondary to cataract surgery and stable rates of endophthalmitis secondary to intravitreal injections. Visual outcomes were bimodal; while many do very poorly, some have good visual recovery.

## 1 Introduction

Endophthalmitis is a severe and sight‐threatening ocular disease characterised by profound intraocular inflammation secondary to bacterial or fungal infection. There is significant variability worldwide regarding the precipitating causes and microbiology of endophthalmitis. Furthermore, there has been a change in the trends of endophthalmitis through the years. These trends have been theoretically influenced by factors such as the introduction of prophylactic antibiotics in cataract surgery, intravitreal injections and changing microbiology [1, 2, 3, 4].

Understanding the trends and changes in the microbiology and precipitating causes of endophthalmitis, as well as their effects on visual outcomes, is key to optimising clinical management. So far, there have been limited studies assessing the longitudinal changes in endophthalmitis characteristics through the years. The two largest and most recent studies are from 10 years from France (2009–2018, *n* = 7522) and 23 years from the USA (2000–2022, *n* = 4305) [5]. However, there has been limited focus on Australian populations, with the largest Australian cohort over 15 years (1998–2013, *n* = 205) [6].

As such, we aim to study the longitudinal trends in the microbiology and precipitating causes of endophthalmitis in Victoria, Australia over a 25‐year period. Furthermore, we aim to investigate the visual outcomes of patients with endophthalmitis across this period. This was, to the best of our knowledge, the longest study of endophthalmitis to date.

## 2 Methods

This study uses data from the Victorian Endophthalmitis Registry, a prospectively maintained database of all endophthalmitis cases presenting to the Royal Victorian Eye and Ear Hospital (RVEEH) in Melbourne, Victoria, Australia, the state‐wide tertiary referral centre for ocular conditions. In the Victorian context, almost all endophthalmitis is managed at the RVEEH. This is partly due to the admitting rights of ophthalmologists; there are few hospitals across the state with overnight beds that ophthalmologists can admit to, and this is the statewide service for emergency vitreoretinal conditions. While there may be an occasional case managed in a general hospital, this thus provides a unique environment to capture nearly all cases of endophthalmitis state‐wide.

This database was created in 1997 and has been prospectively maintained, with medical staff entering patient details at the time of admission. The database is periodically checked for completeness and follow‐up data is recorded. All cases of endophthalmitis at RVEEH are managed by a subspecialist vitreoretinal surgeon.

Endophthalmitis was diagnosed clinically as profound panuveitis caused by likely bacterial or fungal intraocular infection. All cases were reviewed by the vitreoretinal unit and the diagnosis confirmed by the on‐call surgeon or head of unit. Culture‐negative cases were classified as endophthalmitis based on the clinical scenario and presence of polymorphs within the eye. Initial management was guided by protocols. Currently, the standard of care is an aqueous and vitreous tap with intravitreal injection of ceftazidime and vancomycin, usually with dexamethasone. Where feasible, patients with a presenting visual acuity of light perception or worse undergo a vitrectomy with intravitreal therapy as above.

All patients who presented with endophthalmitis between 1 January 1998 and 31 December 2023 were included in this study. Demographic data, examination findings, precipitating causes, clinical risk factors, management and outcomes were collected prospectively. Precipitating causes were categorised into cataract surgery, cornea (corneal infections with secondary endophthalmitis), endogenous endophthalmitis, glaucoma surgery (any acute or delayed complication resulting in endophthalmitis), intravitreal injections, trauma (including corneal lacerations) and vitreoretinal surgery. Microbiological testing was undertaken at St Vincent's Hospital, Melbourne, Victoria, Australia.

Culture positivity was defined as microbiological growth on aqueous or vitreous samples (including taps, biopsies and washings), enucleation or evisceration specimens, and corneal scrapings if microbial keratitis was deemed to be the precipitating cause. Pathology with gram stain positivity but no microbiological growth was considered culture negative as it was not possible to be certain of accuracy. Use of intracameral antibiotics (cefazolin) was introduced as protocol at RVEEH in 2008; as such, 1 January 2008 was used for analysis of the impact of intracameral antibiotics.

Population data of the total number of annual procedures in Victoria were taken from the Medicare Benefits Scheme (MBS) online statistics report.

Data was stored in REDCap data management software, primarily migrated from older database software and checked for accuracy.

Statistical analyses were performed using Stata/IC software version 14 (StataCorp, College Station, TX). Dichotomous outcomes were analysed using the chi2 test. Patients with a follow‐up timeframe of less than 28 days were excluded from follow‐up analyses. A poor outcome was defined as a final visual acuity of less than 6/60 or an enucleation/evisceration. Univariate logistic regression was used to determine risk factors of poor outcomes.

Ethical approval for this study was granted by the Human Ethics Research Committee at RVEEH (HREC approval number 02/468H/20). Research was conducted in accordance with the Declaration of Helsinki.

## 3 Results

**FIGURE 1:**
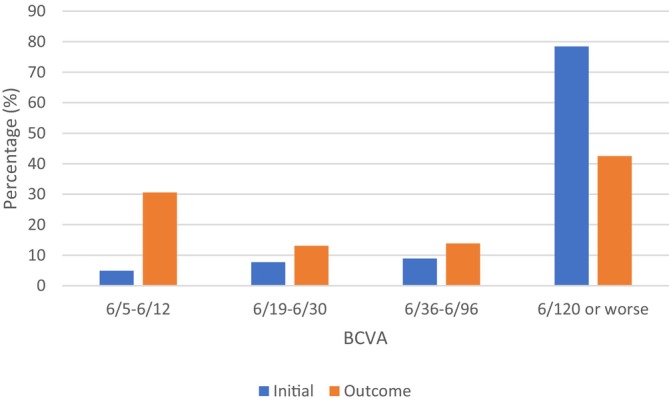
Best corrected visual acuity (BCVA) at presentation and at final follow‐up.

A total of 1423 eyes from 1408 patients were included in this study. The mean (SD) age was 71.35 (16.12) years. The median presenting BCVA was hand movements, with an interquartile range of counting fingers to light perception (Figure 1). At final follow up, 30.6% of patients were 6/12 or better, 13.1% were 6/19–6/30, 13.9% were 6/36–6/96 and 42.5% were 6/120 or worse (Figure 1).

**FIGURE 2:**
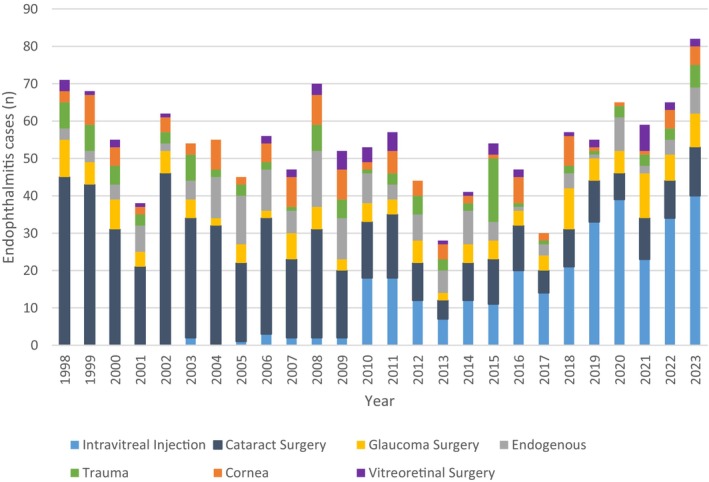
Precipitating causes of endophthalmitis across the 25 years.

The total number of endophthalmitis cases per year has remained relatively stable between 1998–2023 (Figure 2). Across most years, this ranged between 41–71 per annum, with outliers in 2001 (*n* = 38), 2013 (*n* = 28) and 2023 (*n* = 84). There was no overall trend to increasing numbers of cases per year.

The most common precipitating cause across the 25‐year period was cataract surgery (36.79%), followed by intravitreal injections (22.60%) (Table 1). Cataract surgery was the precipitating cause in the majority of yearly cases between 1998–2004 (55.26%–63.38%). It remained the plurality of yearly cases between 2005–2009 (34.62%–54.39%). This decreased to less than 30% since 2010, and less than 20% since 2020 (Figure 2). Between 2005–2009, intravitreal injections comprised 5% or less of endophthalmitis cases. It has risen to become the plurality of causes since 2010, causing 20%–33.33% of yearly cases between 2010–2015. This continued to increase, causing 38.98%–60.00% of yearly cases between 2016–2023. The annual proportions of other causes of endophthalmitis have stayed relatively stable through the years. This included endogenous endophthalmitis (mean annual proportion 12.43%), previous glaucoma drainage surgery (including blebitis) (mean annual proportion 10.61%), corneal pathology (including microbial keratitis and corneal perforations) (mean annual proportion 8.16%), penetrating trauma (mean annual proportion 7.21%) and following vitreoretinal surgery (mean annual proportion 4.49%).

**TABLE 1:** Precipitating causes of endophthalmitis cases.

Precipitating cause	Number (*n*)	Percent
Cataract surgery	521	36.79
Intravitreal injection	320	22.60
Endogenous	161	11.37
Unknown infection	61	37.89
Other infection	50	31.06
Intravenous drug use	39	24.22
Urinary infection	11	6.83
Glaucoma surgery	156	11.02
Corneal pathology	117	8.26
Trauma	88	6.21
Vitreoretinal surgery	48	3.39
Other	6	0.42

**FIGURE 3:**
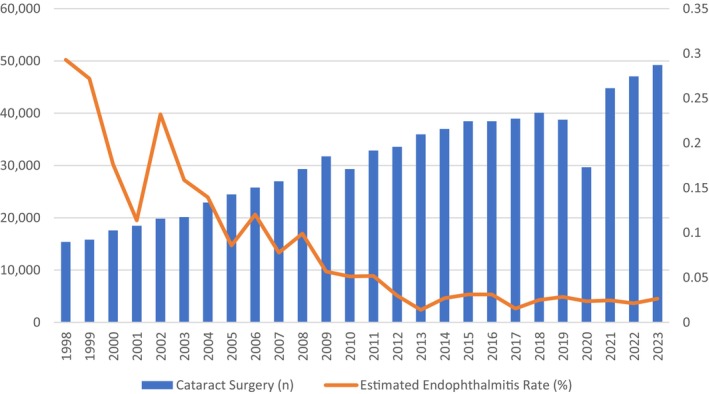
Annual number of cataract surgeries in Victoria compared to estimated endophthalmitis rates.

**FIGURE 4:**
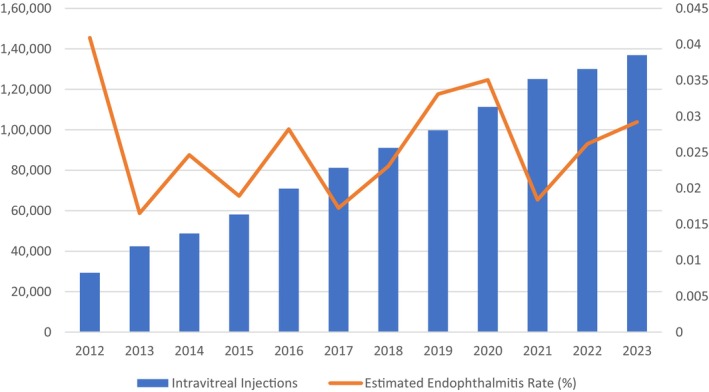
Annual number of intravitreal injections in Victoria compared to estimated endophthalmitis rates.

Between 1998 and 2023, the annual number of cataract surgeries performed in Victoria increased from 15 366 to 49 195 (Figure 3). The annual number of endophthalmitis cases caused by cataract surgery trended downwards from 45 in 1998 to 13 in 2023. Thus, the estimated rate of endophthalmitis secondary to cataract surgery decreased from 0.29% to 0.02%. Conversely, while the annual number of intravitreal injections rose each year from 29 334 in 2012 to 136 907 in 2023, the endophthalmitis rate stayed relatively stable through the years (Figure 4).

Eight hundred and ninety‐seven (63.03%) were culture‐positive and 14 (0.98%) had micro‐organisms identified on gram stain but not cultured; the rest had no microbiological growth. A summary of growth is shown in Table 2. The plurality of samples grew gram positive bacteria (42.94%), of which staphylococcus species was most common (26.42%). *Staphylococcus epidermidis* was the most common organism grown (23.93%), followed by *staphylococcus aureus* (11.03%). Streptococcus species made up 10.54% of results, followed by gram negative organisms (7.87%). Mixed growth comprised 6.89%, and fungi 4.36%.

**TABLE 2:** Summary of microbiology from ocular samples (from culture‐positive ocular samples).

Organism	Number (*n*)	Percentage (%)
Other gram‐positive organisms	367	40.73
Coagulative‐negative staphylococci	327	36.29
Gram negative organisms	121	13.43
Fungi	65	7.21

**FIGURE 5:**
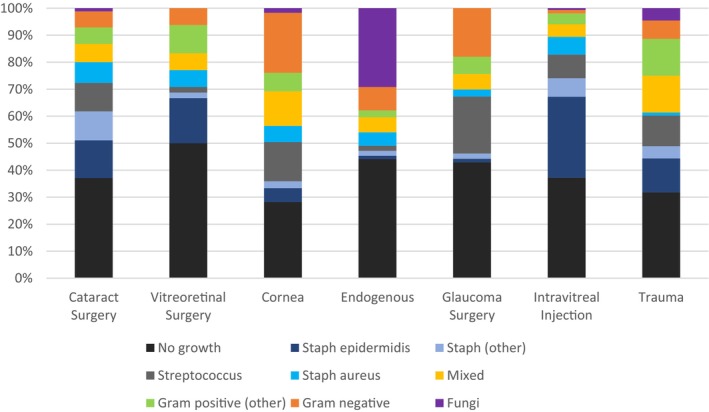
Summary of microbiological growth based on precipitating cause.

There was a statistically significant difference between the microbiology grown within each precipitating cause group (*p* < 0.01). Of the cataract surgery group, 37.04% had no growth, followed by 14.01% growing *staphylococcus epidermidis* and 10.75% growing other staphylococci (Figure 5). In the intravitreal injection group, 37.19% had no growth, 30.00% grew *staphylococcus epidermidis* and 8.75% grew streptococcus species. In the cornea group, 28.21% had no growth, followed by 22.22% gram‐negative growth and 14.53% streptococcal species. In the endogenous group, 44.10% had no growth, followed by 29.19% growing fungi and 8.7% with gram‐negative growth. Of the glaucoma group, 42.95% had no growth, followed by 21.15% growing streptococcal species and 17.95% having gram‐negative growth. In the trauma group, 31.82% had no growth, and 13.64% had other gram‐positive growth or had mixed growth. In the vitreoretinal surgery group, 50.00% had no growth, 16.67% grew *staphylococcus epidermidis* and 10.42% had other gram‐positive growth.

A sub‐analysis was performed of endophthalmitis secondary to cataract surgery, analysing microbiology prior to and following intracameral cefazolin use as a protocol at RVEEH. A total of 522 cases were included, 324 (62.06%) prior to cefazolin (1998–2007) and 198 (37.93%) following cefazolin (2008–2023); this averaged to 32.4 cases per year prior to cefazolin and 13.2 per year following cefazolin. A significant change was found between microbiology overall (*p* < 0.01) (Table 3). There was an overall increase in proportion of culture‐negative cases (31.48% to 45.96%) and gram‐negative cases (4.63% to 8.08%). Despite the increase in proportion of gram‐negative cases, the total number of gram‐negative cases per year decreased from 1.5 to 1.1. There was a decrease in proportion of staphylococcal species overall (36.42% to 25.76%), as well as mixed growth (8.33% to 4.04%).

**TABLE 3:** Comparison of microbiological growth prior to and following intracameral cefazolin use as protocol in cataract surgery.

	Pre‐cefazolin era (1998–2007) (*n* = 324, 62.07%)	Cefazolin era (2008–2023) (*n* = 198, 37.93%)
No growth	102 (31.48%)	91 (45.96%)↑
Staph epidermidis	48 (14.81%)	25 (12.63%)↓
Streptococcus	32 (9.88%)	23 (11.62%)↑
Staph (other)	38 (11.73%)	18 (9.09%)↓
Gram negative organisms	15 (4.63%)	16 (8.08%)↑
Staph aureus	32 (9.88%)	8 (4.04%)↓
Mixed	27 (8.33%)	8 (4.04%)↓
Gram positive organisms (other)	28 (8.64%)	5 (2.53%)↓
Fungi	2 (0.62%)	4 (2.02%)↑

60.2% of cases had 1 tap and inject performed, 37.2% had 2 or more tap and injects performed, 2.1% had a primary enucleation, 0.5% had a primary vitrectomy without prior tap and inject. The mean (SD) number of tap and injects was 1.49 (0.72), with a range of 0 (0.8%) to 5 (0.29%). 348 (24.46%) had a vitrectomy; of those, 117 (33.62%) were more than 24 h following presentation (considered “delayed vitrectomy”). Indications for delayed vitrectomy included ongoing or worsening inflammation, non‐clearing visually‐significant vitreous debris, subretinal abscess and retinal detachment.

In total, 113 (7.94%) had an enucleation or evisceration, of which 29 (25.6% of enucleations) were primary enucleation/eviscerations within 1 day. The indications for primary enucleations included panophthalmitis and large corneal perforations or corneal graft dehiscence which were not amenable to eye‐preserving surgery. The median (IQR) days to enucleation was 5 [2, 7]. Later enucleations were indicated for blind painful eyes. Logistic regression was performed of patient demographics, precipitating cause, use of cefazolin, vitrectomy, delayed vitrectomy and microbiological growth. Corneal infections (OR 10.78, *p* < 0.01) and trauma (OR 2.95, *p* = 0.04) as precipitating factors were associated with enucleation. No growth on culture (OR 0.29, *p* < 0.01), endophthalmitis following intravitreal injections (0.2, *p* = 0.02), and *staphylococcus epidermidis* growth (OR 0.06, *p* < 0.01) were associated with a lower risk of enucleation.

A total of 1193 patients had follow‐up data recorded (83.84%). After excluding patients with less than 28 days of follow‐up (unless they underwent enucleation or evisceration), a total of 923 patients (64.86%) were included in follow‐up analysis. The median (IQR) follow‐up period in days was 113.5 (49, 322). The median final BCVA was 6/60, with an interquartile range of 6/12 to hand movements (Figure 1). Final outcomes were biphasic; 43.6% of patients were 6/30 or better and 56.4% were 6/36 or worse.

**FIGURE 6:**
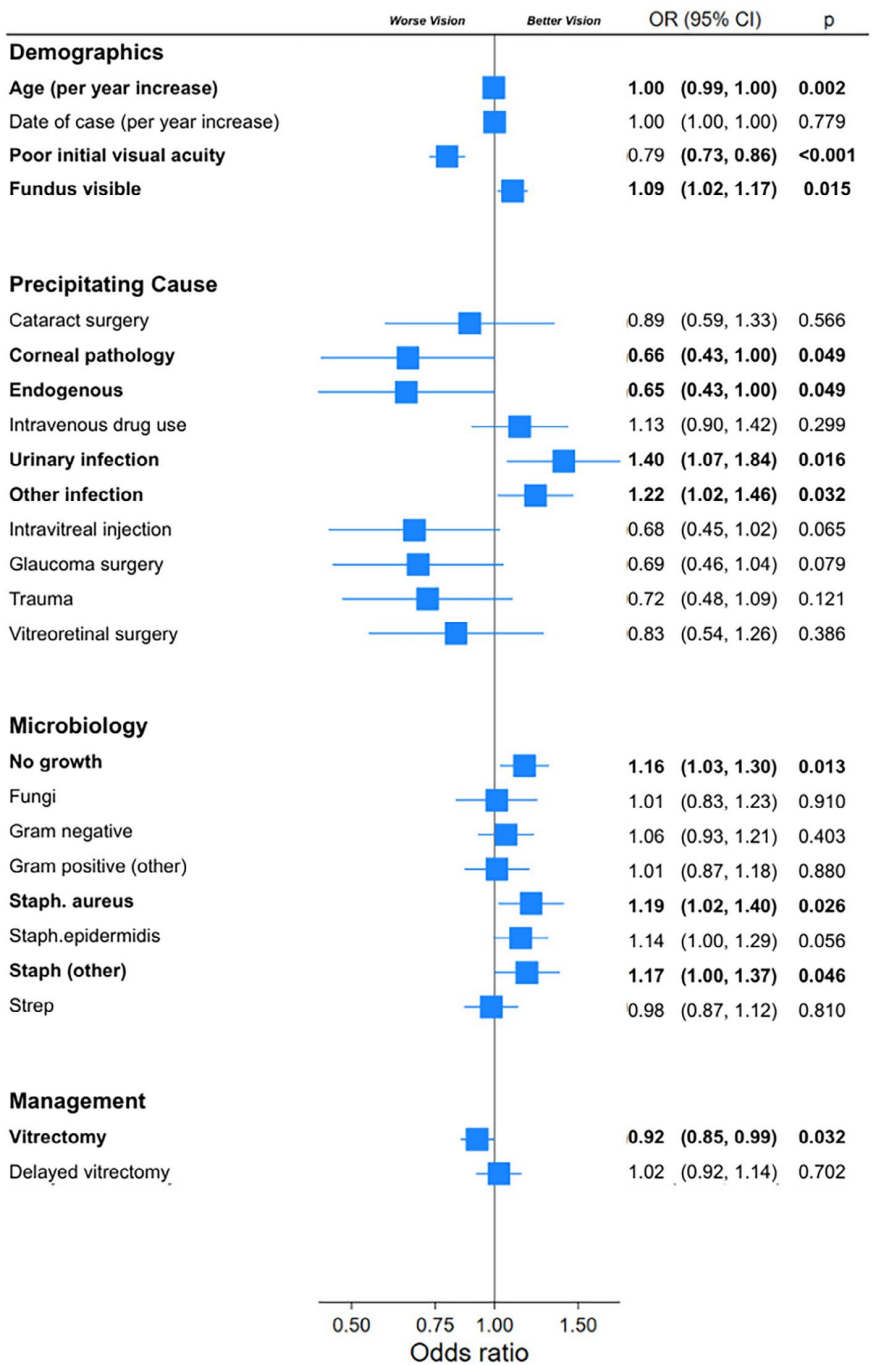
Multivariate logistic regression of factors associated with good outcomes (visual acuity 6/12 or better).

Overall, 30.6% of patients had a good visual outcome, defined as final BCVA 6/12 or better. Logistic regression was performed of patient demographics, precipitating cause, use of cefazolin, vitrectomy, delayed vitrectomy and microbiological growth (Figure 6). Cataract surgery as a precipitating cause was associated with good outcomes (OR 3.96, *p* < 0.01), as was endogenous endophthalmitis secondary to urinary infections (OR 5.97, *p* = 0.02) or other infections (OR 3.35, *p* = 0.04), *staphylococcus aureus* growth on culture (OR 3.18, *p* = 0.03) and no growth on culture (OR 2.57, *p* = 0.02). Factors less associated with good outcomes included increased age (OR 0.98, *p* < 0.01), poor initial VA (OR 0.32, *p* < 0.01) and glaucoma surgery (OR 0.46, *p* = 0.02).

**FIGURE 7:**
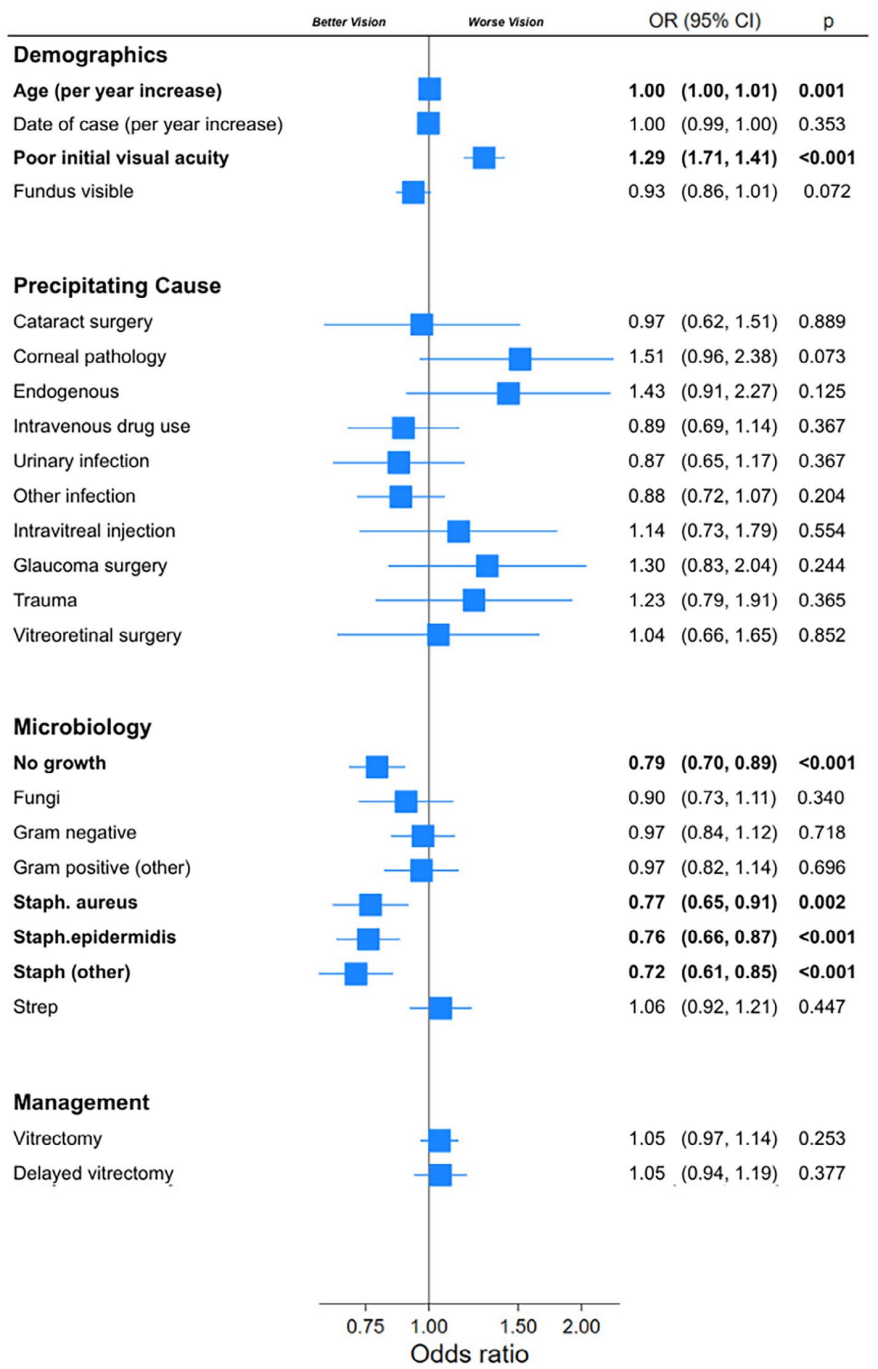
Multivariate logistic regression of factors associated with poor outcomes (visual acuity worse than 6/60 or enucleation/evisceration).

Overall, 413 (44.99%) patients had a poor visual outcome, defined as final BCVA less than 6/60 or enucleation/evisceration. Logistic regression was performed on patient demographics, precipitating cause, use of cefazolin, vitrectomy, delayed vitrectomy, and microbiological growth (Figure 7). Increased age at admission was associated with a poor outcome (OR 1.02, *p* < 0.01), as was presenting VA less than 6/60 (OR 4.96, *p* < 0.01) and corneal infection causing endophthalmitis (OR 2.56, *p* = 0.02). Factors associated with a lower risk of poor visual outcomes included cataract surgery (OR 0.20, *p* < 0.01), intravitreal injection (OR 0.53, *p* = 0.04), and vitreoretinal surgery (OR 0.35, *p* = 0.04). Similarly, infections that were culture‐negative (OR 0.29, *p* < 0.01), or caused by *Staphylococcus aureus* (OR 0.26, *p* < 0.01), *Staphylococcus epidermidis* (OR 0.26, *p* < 0.01), or other staphylococcal species (OR 0.17, *p* < 0.01) were also associated with more favourable outcomes. All other factors were not statistically significant. These remained statistically significant on multivariate analysis.

In total, 41.64% of patients who did not undergo vitrectomy had a poor outcome, compared to 51.23% of those who underwent an early vitrectomy (*p* = 0.03).

## 4 Discussion

This 25‐year period was, to the best of our knowledge, the longest study of endophthalmitis cases and provides a longitudinal perspective of endophthalmitis and its change through the years.

The causes of endophthalmitis were, as expected, primarily cataract surgery between 1998–2004. With the rise of intravitreal injections, they became the plurality of causes since 2011. These findings are comparable to the largest database study from France; here, they demonstrated an overall incidence of endophthalmitis as 0.05% from 2009–2018, with a decreasing incidence of endophthalmitis following cataract surgery and a stable incidence of endophthalmitis following intravitreal injections, despite increasing numbers of injections [5]. The rates of all other causes remained relatively stable across the years. This is in keeping with other Australian studies, as well as data from Israel, Norway and Brazil [8, 9, 10, 11]. This is, however, in contrast to countries such as Columbia, China and the Philippines, where trauma was the main cause of exogenous endophthalmitis, comprising up to 66.9% of exogenous causes [12, 13, 14]. The rate of endophthalmitis in cataract surgery decreased from 0.29% to 0.02% across the 25‐year period. This is in keeping with the largest US study, which demonstrated a decrease in endophthalmitis rate following intraocular procedures from 0.2% in 2000 to 0.05% in 2022 [15].

63.03% of cases were culture‐positive in this cohort. Laboratory analysis was based on gram stain and culture growth. Reviewing techniques to better improve identification of pathogens, such as polymerase chain reaction (PCR) and other emerging molecular techniques, is important to better identify organisms and their optimal treatment [4].

Staphylococcal species was the most common microbiology grown (26.42%), followed by streptococcal species (10.54%) and gram‐negative organisms (7.87%). Growth in the cataract and intravitreal injection groups followed this trend. In the endogenous endophthalmitis group, however, the plurality grew fungal elements, followed by gram‐negative growth.

Staphylococcal species is widely acknowledged to be the most common microbiological cause of endophthalmitis. Gram‐positive organisms, primarily staphylococcus and streptococcus, were the most common organisms found in studies in the USA, China and Brazil [3, 10, 14, 16, 17]. This was in contrast to studies from India, which saw higher rates of gram‐negative organisms and fungal growth, and the Philippines, which had lower rates of gram‐positive infections but higher rates of mixed organisms [13, 18].

In Australia, the largest study prior to this, a 15‐year study performed in Queensland demonstrated *staphylococcus epidermidis* (23.1%), coagulase‐negative staphylococcus (12.7%) and streptococcus viridans (10.0%) were the most common species [6]. However, candida and fungal mould grew in 6.1% and 5.7% respectively, which was significantly higher than our overall rates of fungus (4.36%), possibly due to the warmer and more humid climate seen in parts of Queensland [19].

Logistic regression demonstrated that staphylococcal species were significantly less likely to have poor outcomes (OR 0.17–0.26, *p* < 0.01); staphylococcal epidermidis was less likely to require enucleation (OR 0.62, *p* = 0.01) and staphylococcal aureus was more likely to have good visual outcomes (OR 3.18, *p* = 0.03). This has been extensively reported previously [5, 9, 20, 21, 22]. Staphylococcal species were most commonly grown in endophthalmitis secondary to cataract surgery and intravitreal injections, in keeping with previous studies [7, 20, 22, 23, 24]. It can be thus extrapolated that these two groups of endophthalmitis were more likely to be more indolent than other causes, which has been shown in the literature [25, 26, 27, 28, 29, 30, 31].

Overall, the number of endophthalmitis cases secondary to cataract surgery per year decreased following the introduction of intracameral cefazolin use as protocol in cataract surgery (an average rate of 0.16% between 1998–2007 compared to 0.03% per year between 2008–2023). While it was only protocol at RVEEH, a state‐wide survey of ophthalmologists in 2013 found that 84.4% used routine intracameral antibiotics in private surgery, the majority of which being intracameral cefazolin [32]. Given the rise in cataract surgery through the years, this decline is understated in our data. Endophthalmitis rates following cataract surgery decreased from 0.29% to 0.02% across our study period (Figure 3), dropping to equal to or lower than reported worldwide, such as from France (0.12%–0.16%), Sweden (0.02%) and USA (0.08%–0.11%) [5, 15, 33, 34]. This is of note given international use of cefuroxime or moxifloxacin; the use of prophylactic intracameral cefazolin commenced in 2007 due to drug availability. This is in keeping with the current literature supporting prophylactic antibiotic use in cataract surgery [35, 36]. Furthermore, a significant change in microbiology was found between both periods. There was an increase in culture‐negative cases (31.48% to 45.96%, *p* < 0.01); this once again corresponds with less aggressive cases of endophthalmitis. This was accompanied by a decrease in staphylococcal species (36.42% to 25.76%, *p* = 0.01) but a statistically insignificant increase in the proportion of gram‐negative cases (4.63% to 8.08%, *p* = 0.1). This is in keeping with the antimicrobial coverage of cefazolin, with good gram‐positive cover but limited gram‐negative cover. However, despite the increase in the proportion of gram‐negative cases, the average number of cases per year decreased from 1.5 (across the pre‐cefazolin period) to 1.1 (following routine cefazolin use); as such, while the proportion of gram‐negative growth has increased, total numbers have not and this thus does not support increasing prophylactic gram‐negative cover.

The rate of streptococcal growth in intravitreal injections (overall 8.75%) was less than in cataract surgery (overall 10.56%). This is in keeping with newer data; Ong et al. demonstrated lower rates of streptococcal growth in IVIs compared to cataract surgery in 2019 [23]. This is in contrast to earlier reports of streptococcal infection being more common in IVIs [37]. It is possible that this was secondary to increasing respiratory precautions including mask use and limiting conversation, thereby decreasing respiratory pathogen spread [38]. While our results did not demonstrate a trend to reducing over time, it is possible this was due to small numbers overall.

Endophthalmitis due to corneal pathology was found to be most strongly associated with enucleation (OR 10.78, *p* < 0.01), as has been previously described [39, 40, 41]. The two commonest organisms grown were gram‐negative organisms (22.22%) and streptococcal species (14.53%). While our study did not reach statistical significance in these organisms being related to poor outcomes, previous studies have demonstrated poor outcomes with both species [21, 22, 42, 43, 44, 45, 46]. Similarly, endophthalmitis secondary to a complication of glaucoma procedures had commonest organisms being streptococcal species (21.15%) and gram‐negative organisms (17.95%). Nevertheless, glaucoma procedures were not associated with higher risks of poor outcomes in our cohort. This thus suggests reasons beyond organisms, including corneal scarring and possible perforations, that lead to poor outcomes in patients with corneal pathology.

Endophthalmitis following trauma was associated with greater likelihood of enucleation/evisceration (OR 2.95, *p* = 0.04). Current clinical practice at RVEEH is oral fluoroquinolones (ciprofloxacin during the duration of the study; recently changed to moxifloxacin) following penetrating trauma. Fluoroquinolones have been found to provide a 90% minimum inhibitory concentration in the vitreous cavity against gram‐negative and selected gram‐positive bacteria; newer agents such as moxifloxacin have greater coverage against gram‐positive bacteria such as staphylococcus and streptococcus [47]. The most common microbiology in our cohort was non‐staphylococcal gram‐positive bacteria or mixed growth; this was reflected in a recent meta‐analysis [48].

Endophthalmitis secondary to vitreoretinal surgery had the highest proportion of culture‐negative samples (50.00%), followed by *staphylococcus epidermidis* (16.67%) and other gram‐positive growth (10.42%). Vitreoretinal surgery as a precipitating factor was found to be less likely associated with poor outcomes (OR 0.35, *p* = 0.04); one contributing factor is culture negativity and *staphylococcus epidermidis* were both significantly less likely to be associated with poor outcomes. Recent studies have demonstrated that post‐vitrectomy endophthalmitis has favourable outcomes. Another single‐centre study demonstrated similar outcomes between post‐vitrectomy and IVI endophthalmitis, with less virulent organisms found in the post‐vitrectomy cohort [49]. This is opposite to other studies which have demonstrated poor outcomes from vitreoretinal surgery [25, 50, 51]. While it is unclear why vitreoretinal surgery had better outcomes in our cohort, possible explanations include close follow‐up post vitrectomy, the removal of vitreous as a growth medium for bacteria, and effects of the tamponade on bacterial spread and intravitreal antibiotic penetration [21, 25].

Overall, outcomes were poor. The median final BCVA was 6/60, with 44.99% of patients having vision worse than 6/60 or an enucleation or evisceration. Nevertheless, there was a biphasic pattern to visual outcomes, with 30.6% of patients reaching 6/12 or better; this demonstrated that while many patients had poor visual outcomes, a significant proportion reached good outcomes (Figure 1). Logistic regression demonstrated increased age at admission (OR 1.02, *p* < 0.01), corneal infection (OR 2.56, *p* = 0.02) and poor presenting BCVA (less than 6/60) (OR 4.96, *p* < 0.01) were associated with poor outcomes. Good visual outcomes (BCVA 6/12 or better) were associated with no growth on culture (OR 2.57, *p* = 0.02). Culture negativity has been previously extensively described as having better outcomes [20, 21, 22]. This may be due to less virulent bacteria, earlier diagnosis or lesser intraocular bacterial load.

However, factors including gram‐negative organisms and vitrectomy (including delayed vitrectomy) were not found to affect outcomes. As discussed above, gram‐negative organisms and streptococcal species had been previously described as having poor outcomes. It is possible that due to the small sample size of these organism groups we were unable to reach statistical significance. Patients who underwent early vitrectomy had poorer outcomes than those who did not undergo vitrectomy (51.23% vs. 41.64%); this did not change on sub‐analysis of poorer visual acuity. Given patients were not randomly allocated, and thus selected for vitrectomy based on the severity of disease, the impact on vision is difficult to assess in this cohort.

The main strength of this study was that it was, to the best of our knowledge, the longest longitudinal study of endophthalmitis. This prospective study enabled a clear understanding of general patterns of microbiology and causes, as well as providing a large dataset to analyse factors affecting poor outcomes. Furthermore, as the RVEEH is the tertiary referral centre for state‐wide ocular infections, almost all cases of endophthalmitis across Victoria are treated here. This study was thus able to provide an overview of almost all cases of endophthalmitis across a state within the 25‐year timeframe.

The limitations of this long study period are the missing data; data not entered at the time of the infection was difficult to retrieve for patients in earlier years of the study. Furthermore, due to the state‐wide referral service, many patients were followed up externally or privately. While all attempts were made to collect data from external sources, this ultimately resulted in missing follow‐up data, thereby potentially affecting analysis of outcomes. Additionally, there are limitations in the calculation of endophthalmitis rates. While the RVEEH captures almost all Victorian cases of endophthalmitis, an occasional case may be managed in a general hospital. Furthermore, as our data of intravitreal injections come from the Medicare item numbers, this may be under‐reported. Ultimately, while it is possible to have mild underestimates in rates, we believe we collected almost all cases of endophthalmitis across Victoria due to being the state‐wide service for emergency vitreoretinal cases.

Overall, this study provided an overview of almost all cases of endophthalmitis across Victoria, Australia, in a 25‐year period. We demonstrated that culture‐negative endophthalmitis and staphylococcal species, while the most common causes of endophthalmitis, were associated with better outcomes, and predisposing corneal pathology, associated most strongly with gram‐negative organisms and streptococcus, were associated with poor outcomes. The change in microbiology profiles of endophthalmitis following the introduction of prophylactic cefazolin, with greater proportions of culture‐negative and gram‐negative growth and decreased proportions of staphylococcal species, was in keeping with known antimicrobial coverage of cefazolin. Finally, while overall outcomes were poor, with a median BCVA of 6/60 post‐endophthalmitis, 30.6% reached 6/12 or better, demonstrating it is possible to have good recovery from endophthalmitis in some circumstances.

## Funding

The authors have nothing to report.

## Conflicts of Interest

The authors declare no conflicts of interest.

## Data Availability

The data that support the findings of this study are available from the corresponding author upon reasonable request.
